# Decoding the direction of imagined visual motion using 7 T ultra-high field fMRI

**DOI:** 10.1016/j.neuroimage.2015.10.022

**Published:** 2016-01-15

**Authors:** Thomas C. Emmerling, Jan Zimmermann, Bettina Sorger, Martin A. Frost, Rainer Goebel

**Affiliations:** a Department of Cognitive Neuroscience, Maastricht University, 6229 EV Maastricht, The Netherlands; b Maastricht Brain Imaging Center, 6229 ER Maastricht, The Netherlands; c Netherlands Institute for Neuroscience (NIN), 1105 BA Amsterdam, The Netherlands

**Keywords:** Decoding, Functional magnetic resonance imaging, Multi-voxel pattern analysis, Ultra-high field MRI, Visual mental imagery

## Abstract

There is a long-standing debate about the neurocognitive implementation of mental imagery. One form of mental imagery is the imagery of visual motion, which is of interest due to its naturalistic and dynamic character. However, so far only the mere occurrence rather than the specific content of motion imagery was shown to be detectable. In the current study, the application of multi-voxel pattern analysis to high-resolution functional data of 12 subjects acquired with ultra-high field 7 T functional magnetic resonance imaging allowed us to show that imagery of visual motion can indeed activate the earliest levels of the visual hierarchy, but the extent thereof varies highly between subjects. Our approach enabled classification not only of complex imagery, but also of its actual contents, in that the direction of imagined motion out of four options was successfully identified in two thirds of the subjects and with accuracies of up to 91.3% in individual subjects. A searchlight analysis confirmed the local origin of decodable information in striate and extra-striate cortex. These high-accuracy findings not only shed new light on a central question in vision science on the constituents of mental imagery, but also show for the first time that the specific sub-categorical content of visual motion imagery is reliably decodable from brain imaging data on a single-subject level.

## Highlights

• Four different directions of visual motion can be decoded during imagery at 7 T.
• We found very high classification accuracies in single subjects.
• High variability between activation patterns and accuracies of different subjects

## Introduction

The nature and exact representation of complex mental imagery in the human brain is recognized as one of the keys to understanding creativity and intelligence (Ferguson, 1977, Kozhevnikov et al., 2013). If neuroimaging studies were able to reveal the neural correlates of mental imagery with a similar degree of accuracy as for perception, this may potentially allow for a better understanding of human creativity and intelligence since mental imagery seems to be a prerequisite for both (Miller, 1996).

One particularly naturalistic form of imagery is the imagination of visual motion. Visual motion is a crucial domain in daily life; the processing of visual motion is not only necessary to maintain a coherent percept of one's surrounding but also enables us to separate objects from each other and perceive depth. So far, research in cognitive neuroscience has mostly focused on static mental images such as orientations (Albers et al., 2013, Harrison and Tong, 2009), shapes (Stokes et al., 2009, Stokes et al., 2011), objects (Cichy et al., 2012, Lee et al., 2012, Reddy et al., 2010), and scenes (Johnson and Johnson, 2014). Investigating motion imagery on the other hand goes beyond static and towards dynamic imagery processes and could, thus, enable a better understanding of sustained forms of imagery. Due to its naturalistic character, motion imagery is close to and directly translatable to actual action implementation, for instance in human machine interfacing. While neural activation patterns during visual short-term memory of perceived visual motion have been shown recently (Emrich et al., 2013), scientific knowledge about neural correlates of motion imagery is still limited. This is due to the inherently private nature of imagery and inter-individual differences in personal strategies (Kozhevnikov et al., 2002, Kozhevnikov et al., 2005, Poltrock and Brown, 1984; also note the recently described condition of congenital aphantasia: Zeman et al., 2015) and brain areas (Kosslyn et al., 2001, Motes et al., 2008, Wraga et al., 2003) recruited during imagery. In addition, so far only the occurrence (categorical decoding), but not the specific content (sub-categorical decoding) of motion imagery has been decodable (Goebel et al., 1998) and the question of whether the actual specific content of imagined motion is decodable, is still to be answered.

When it comes to the neural correlates of imagery, numerous findings show that perception and imagery share neural circuits (for a review see Kosslyn et al., 2006). This suggests that also brain regions involved in visual motion imagery overlap with those involved in visual motion perception. The most important motion-responsive brain areas include direction-selective cells in V1 (Hubel and Wiesel, 1968, McLean and Palmer, 1989, Movshon et al., 1978), human area V3A (hV3A) (Tootell et al., 1997, Van Oostende et al., 1997), and the human motion complex (Cheng et al., 1995; hMT +; Dupont et al., 1994, Tootell et al., 1995). Different directions of perceived visual motion can be decoded from several of these areas in the visual cortex (Kamitani and Tong, 2005). More recently, the axis-of-motion selective columnar structure in the middle temporal area (MT) that is known from animal studies (Albright, 1984a, Albright et al., 1984, Diogo et al., 2003, Dubner and Zeki, 1971) was shown in humans using 7  Tesla (T) ultra-high field functional magnetic resonance imaging (fMRI; Zimmermann et al., 2011). In light of these previous studies, V1, V3A, and hMT + are the most likely candidates for the decoding of visual motion imagery.

In order to optimally decode information regarding imagery within these regions, high-field imaging and multivariate analyses seem promising. The recent developments of 7 T ultra-high field fMRI for humans has brought improvements in spatial specificity (Uğurbil et al., 2003, Uludağ et al., 2009) and increases in signal-to-noise ratio (SNR; Vaughan et al., 2001) over conventional 3 T fMRI. Additionally, with higher magnetic field strengths the influence of large draining veins on the blood-oxygen level dependent (BOLD) signal becomes smaller, while the signals from small vessels and capillaries become better detectable (Shmuel et al., 2007). This further enhances the functional specificity. Seminal studies have leveraged these benefits to explore the functional organization of the visual system at the level of cortical columns (Cheng et al., 2001, Goodyear and Menon, 2001, Menon et al., 1997, Yacoub et al., 2007, Yacoub et al., 2008). These structures are well known from animal studies (Bonhoeffer and Grinvald, 1991, Hubel and Wiesel, 1968, Mountcastle, 1957, Tanaka, 1996) and seem to be a crucial functional unit providing a key to understand computational mechanisms in early sensory areas and beyond. In addition to hardware advancements, new data analysis techniques like multi-voxel pattern analysis (MVPA; for an introduction see Pereira, Mitchell, and Botvinick, 2009) and information-based functional brain mapping (Kriegeskorte et al., 2006) enable the extraction of more spatially distributed information from fMRI data. However, there is an ongoing debate on the spatial scale of the signals that MVPA picks up. There are controversial findings on whether large-scale biases can explain decoding performances of MVPA decoding studies (Op de Beeck, 2010a) rather than signals from a fine-grained columnar organization of cortex (Swisher et al., 2010).

Together, ultra-high field fMRI and new analysis algorithms allow one to overcome the resolution boundaries given by conventional neuroimaging methods. Here we apply these methodological advances to the study of visual motion imagery.

The current study aims to decode the specific content of visual motion imagery, accounting for inter-individual differences in imaging strategies and neural activation patterns. In this way, we combine the described advantages of high-field brain imaging and multivariate analyses to decode not simply the occurrence (categorical decoding), but the specific content (sub-categorical decoding) of self-generated motion imagery. Mental imagery during the experiment was well controlled (participants were trained over multiple sessions with the imagery task) and as neutral as possible (the type of visual imagery was chosen to be abstract enough to not interfere with personal memories *etc*.), while still being completely self-generated (*i*.*e*., the experimental paradigm did not include any visual stimulation that could elicit bottom–up activations of the visual system or reactivation following *e*.*g*. a motion stimulus of a previous trial). Therewith we aimed to trigger low-level neural correlates of motion imagery and, thus, expected decodable information on the imagined motion direction in early visual areas (Kamitani and Tong, 2006) and MT (Goebel et al., 1998). Furthermore, in an exploratory way we attempted to link inter-individual differences in the brain activation patterns recruited during motion imagery to individual cognitive imagery styles assessed with self-report questionnaires.

## Materials and methods

### Subjects

15 healthy fMRI-experienced subjects (six females; age: 27.4 ± 6.3 years) with normal or corrected-to-normal vision volunteered in this study. They gave written informed consent and were paid for their participation. All procedures were conducted with approval from the local Ethical Committee of the Faculty of Psychology and Neuroscience at Maastricht University. Two subjects had to be excluded from the analyses because no significant activations could be detected based on a localizer scan (see below). One subject was excluded due to discomfort during the scanning resulting in problems to follow the experimental instructions. All subjects were students or employees at Maastricht University.

### Procedure

In the experiment, subjects had to imagine dots that moved in one of four directions. Subjects were pseudo-randomly assigned to one of two groups. The first group imagined motion directions left, right, up, and down whereas the second group imagined the four diagonal motion directions. Subjects heard a cue on which specific motion direction they should imagine during the upcoming trial. These cues were auditorily presented numbers (1–4) and were associated to the four motion directions in a counterclockwise (subjects 1–9) or clockwise order (subjects 10–15). The orientation of this number-motion direction association (*e*.*g*., “1” = right *vs*. “1” = up) was systematically varied across subjects.

### Training sessions

All subjects attended three training sessions during the week before the scanning session. In each training session subjects followed an adaptive visual imagery training (one run in the first session, two runs in the remaining sessions; see below) and completed two runs of the experimental task (see below) with shortened resting periods. Each training session lasted between 30 and 45 min.

### Adaptive visual imagery training

Coherently moving white dots were presented on the screen together with an auditory cue and faded out after 4000 ms leaving only a white fixation dot. When indicated by a change of fixation color, subjects were instructed to imagine the faded stimulus as vividly as possible. They were asked to press a button as soon as they had a clear picture or “movie” of the stimulus in their mind and to hold the button down for as long as they “saw” this imagined stimulus. If subjects released the button before 8000 ms had passed the stimulus faded in again and they could try again by pressing the button, which faded the stimulus out. After successfully imagining the stimulus for more than 8000 ms the trial ended and was followed by a 5-point Likert scale (1: “No image at all, you only ‘know’ that you are thinking of the object” to 5: “Perfectly clear and vivid as normal vision”) to indicate the clearness of the imagery in the preceding trial. After the adaptive visual imagery training the subjects reported the used imagery strategies to the experimenter.

### Experimental task

**Fig. 1:**
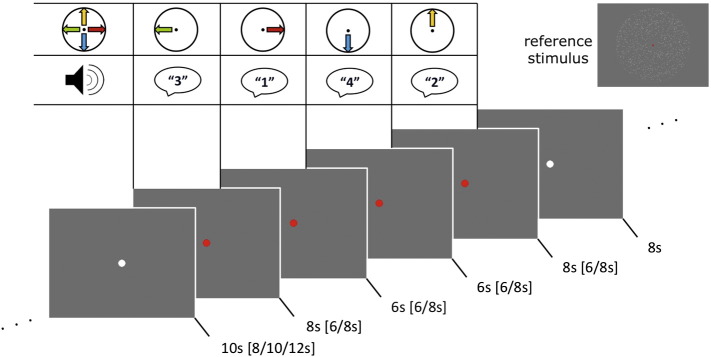
Experimental task. Visual stimulation is shown exemplarily for one block of four trials (red fixation dots) with preceding and succeeding resting blocks (white fixation dots). Durations are shown below each screenshot (possible jittered duration are given in brackets). In the table at the top the motion directions with their corresponding audio cues (that were played to the subject at the start of a trial) are shown (number-motion direction association varied between subjects). At the top right one frame of the reference stimulus is shown representing the reference phase.

In the beginning and in the middle of each run, moving dots were presented as in the training task together with a white fixation dot. This was done I order to facilitate the subjects' recall of the precise stimulus configuration and the number-motion direction association. The four motion directions that had to be imagined by each group were presented for 4000 ms each together with the associated auditory cue (read-out number; from “1” to “4”). After this reference phase and a resting period of 12 s the first block of four trials started (see Fig. 1). We chose to present trials in blocks to maximize the number of trials while allowing subjects to recover from the cognitively demanding task of motion imagery during resting periods. When the fixation dot turned red and the first auditory cue was presented the subjects had to start to imagine the associated motion direction as vividly as possible. The following trials were indicated by the presentation of auditory cues and the subjects had to start to imagine the associated motion direction immediately. Trials lasted for six or eight seconds. The end of the last trial in a block (and thus the cue to stop all imagery) was indicated by the fixation dot turning white again. After a resting period of eight seconds, a 5-point Likert scale was presented. Subjects indicated the clearness of the imagery in the preceding trial block by moving an arrow *via* button press. After another resting period with a jittered duration of eight, ten, or twelve seconds the next block started. Subjects were instructed to fixate the fixation dot throughout the whole experiment. Each run consisted of ten blocks and the subjects completed four runs in the scanner (total duration of experimental runs approximately 40 min; 40 trials per condition).

### Stimuli

Visual stimulation was created with PsychoPy (version 1.78.01; Peirce, 2007) and, in the scanning session, projected on a frosted screen at the top end of the scanner bed using an LCD projector (Panasonic, No PT-EZ57OEL; Newark, NJ, USA). Responses were collected through an MR compatible button box (Current Designs, 8-button response device, HHSC-2 × 4-C; Philadelphia, USA).

### Vividness of Visual Imagery Questionnaire

After recruitment (before the training sessions) subjects filled in the Vividness of Visual Imagery Questionnaire (VVIQ; Marks, 1973). This self-report questionnaire measures subjective clearness and vividness of imagined objects and scenes with 16 items. The vividness ratings for each imagined item are given on a scale from 1 (“No image at all, you only ‘know’ that you are thinking of the object”) to 5 (“Perfectly clear and vivid as normal vision”). After scanning subjects filled in the VVIQ for a second time.

### Object-Spatial Imagery and Verbal Questionnaire

After the scanning session, subjects were contacted to fill out the Object-Spatial Imagery and Verbal Questionnaire (OSIVQ; Blazhenkova and Kozhevnikov, 2009). The OSIVQ is a self-report questionnaire consisting of three scales for “object”, “spatial”, and “verbal” cognitive styles during mental imagery measured by 15 items each. In each item a statement is rated on a scale from 1 (“totally disagree”) to 5 (“totally agree”). We calculated the score on each scale for every subject as described in the original paper (Blazhenkova and Kozhevnikov, 2009).

### Eye-Tracking

To check for eye-movements related to the different directions of imagined motion we recorded eye movements during the scanning session for four subjects (9–12) using an MR-compatible eye-tracker (Real Eye Nano; Avotec, Inc.; Stuart, FL, USA). Eye-tracking data were analyzed using custom code in MATLAB (version 2013a; The MATHWORKS Inc., Natick, MA, USA), code from the “EYE-EEG extension” toolbox (Dimigen et al., 2011; http://www2.hu-berlin.de/eyetracking-eeg) to detect saccades based on the algorithm by Engbert and Mergenthaler (2006), and code from the CircStat toolbox (Berens, 2009). To assess the statistical similarity of saccade directions across trials with different imagined directions we used a non-parametric multi-sample test for equal median directions as implemented in the CircStat toolbox under the null hypothesis that saccade directions did not differ between different directions of imagined motion. We also computed the circular–circular correlation between the direction of imagined motion and the saccade direction and tested it for significance. Furthermore, we analyzed the raw eye gaze position data during trials. After discarding data points during eye blinks (pupil aspect ratio lies outside a confidence interval of ± 2SD around the average pupil aspect ratio in each trial) we fitted a Minimum Volume Enclosing Ellipse (Moshtagh, 2005) to the XY data of each trial. Then we statistically tested the similarity of the ellipse rotations across trials and the circular–circular correlation with the different imagined directions employing the same methods we used for the saccade data (see above).

### Scanning session

First, each subject completed a short 4-block practice version of the experimental task outside the scanner. At the beginning of the scanning session, we recorded an hMT + localizer scan as in Zimmermann et al. (2011) followed by a population receptive field (pRF) retinotopy mapping scan (Dumoulin and Wandell, 2008).

### MRI acquisition

Images were acquired with a Siemens MAGNETOM 7 T scanner (Siemens; Erlangen, Germany) and a 32-channel head-coil (Nova Medical Inc.; Wilmington, MA, USA).

An anatomical dataset was acquired with a T1-weighted magnetization prepared rapid acquisition gradient echo (3D-MPRAGE) sequence (256 sagittal slices, matrix = 384 × 384, voxel size = 0.6 × 0.6 × 0.6 mm^3^). To correct for intensity inhomogeneities an additional gradient echo proton-density (GE-PD) dataset (same parameters as 3D-MPRAGE) was acquired subsequently.

**Fig. 2:**
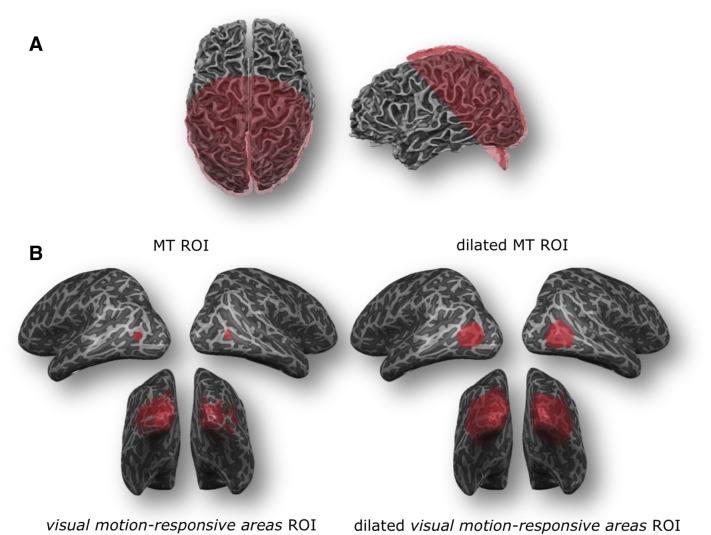
Functional field of view (FOV) and region of interest (ROI) definitions on cortical surfaces. (A) The FOV is shown exemplarily in subject 2 for the functional images (red) from two perspectives (left: top view; right: left-hemispheric view). (B) The MT ROI and the *visual motion*-*responsive areas* ROI are exemplarily shown in the normal (left) and dilated (right) versions in subject 2.

High-resolution functional images were obtained using gradient echo (T2* weighted) echo-planar imaging (EPI) with the following parameters: echo time (TE) = 23 ms, repetition time (TR) = 2000 ms, generalized autocalibrating partially parallel acquisitions (GRAPPA) g-factor = 2, multi-band factor = 2, flip angle = 70°, number of slices = 54, matrix = 130 × 130, voxel size = 1.1 × 1.1 × 1.1 mm^3^. The field-of-view included occipital, temporal, and parietal areas but did not cover large parts of frontal cortex (see Fig. 2A). To correct for EPI distortions additional functional volumes (five volumes in the encoding direction and five volumes with a reversed encoding direction) were acquired right after the GE-PD dataset.

### Imaging data preprocessing

Functional and anatomical images were analyzed using BrainVoyager QX (version 2.8; Brain Innovation; Maastricht, The Netherlands), custom code in MATLAB (version 2013a; The MATHWORKS Inc.; Natick, MA, USA), and PyMVPA (version 2.3; Hanke et al., 2009). Anatomical images were corrected for bias field inhomogeneities by dividing the 3D-MPRAGE images by the GE-PD images and interpolated to a nominal voxel size of 0.55 mm isotropic to match a multiple of 2 of the resolution of the functional data. The detection of the white/gray matter boundary was conducted with the largely automatic segmentation tools of BrainVoyager QX. These tools perform a region-growing method that analyzes intensity histograms and subsequently correct topological errors in the detected borders to finally reconstruct the cortical surfaces (Goebel et al., 2006, Kriegeskorte and Goebel, 2001).

In order to correct for distortions in the echo-planar images we recorded 5 functional volumes of normal and reversed phase encoding. In these pairs of images distortions go in opposite directions and we used them to estimate the susceptibility-induced off-resonance field using a method similar to that described in Andersson et al. (2003) as implemented in FSL (Smith et al., 2004). After performing 3D rigid body motion correction of the remaining functional runs (aligning all subsequent runs to the first functional run) the estimated off-resonance field was used to correct for EPI distortions. Furthermore, functional data were high-pass filtered using a general linear model (GLM) Fourier basis set of two cycles sine/cosine per run (including linear trend removal). Functional runs were co-registered to the individual anatomical scan with an affine (9 parameter) transformation.

### Region-of-interest definition

Regions of interest (ROIs) were then defined using data from the hMT + localizer scan and the pRF retinotopy projected onto an inflated surface reconstruction. Area MT was distinguished from MST in the hMT + complex (Huk et al., 2002, Zimmermann et al., 2011) by thresholding the respective contrast at p < 0.001 and restricting the resulting activation patch to 150 vertices per hemisphere (for creating the MT ROI). A second ROI was created by applying the same contrasts but thresholding at p < 0.05 and including all active patches in visual cortices (for creating the “visual motion-responsive areas” ROI) in each hemisphere. For extended versions of these ROIs the patches were dilated by a static factor (adding neighbor vertices at the patches' boundaries; 20-fold dilation for the MT ROI, 10-fold dilation for *visual motion*-*responsive areas* ROI) to include possibly contributing neighboring regions (see Fig. 2B and Table 2). Areas V1 through V4 were delineated in the polar angle map of the surface-based pRF analysis in both hemispheres. All surface patches were transformed back into volume space (from − 1 mm till + 3 mm from the gray/white matter segmentation boundary) to create the final volume ROIs.

### Multi voxel pattern analyses

Each experimental run was z-scored to eliminate signal offsets and variance differences between runs. After masking the experimental data with the individually defined ROIs the data were split into training and testing datasets. We employed a leave-one-run-out splitting procedure to be able to cross-validate the classification performance. For each split the 1000 voxels (within the respective ROI) with the highest F-values in the respective training data were selected as features. The F values were computed as the standard fraction of between and within class variances (omnibus test). This was done to reduce the high number of voxels within ROIs in 7 T fMRI scans and in order to keep the number of features constant between subjects and ROIs. For each voxel and each trial we extracted the average of 6 s (3 TRs) as features. Averages were computed in a time window from 4 to 10 s after trial onset. The extracted features were then used for an one-vs-one 4-class classification (predicted classes were chosen based on the maximum number of votes in all binary classifications) using a linear support vector machine (SVM; LIBSVM implementation in PyMVPA; Chang and Lin, 2011). We repeated the whole analysis 1000 times with scrambled labels to obtain a distribution under the null hypothesis and tested the probability of the real classification accuracies against this distribution. To assess a group level statistic we tested the real classification performances of each subject against the individual average permutation classification accuracy by means of a Wilcoxon Signed-Ranks test. We chose a non-parametric test as a normal distribution of the decoding accuracies cannot be assumed. Furthermore, we computed Spearman rank-order correlations between mean classification accuracies (across all ROIs) and the OSIVQ questionnaire scores (object, spatial, and verbal scales). We tested these correlations for significance after an FDR-correction for multiple comparisons to reveal possible influences of cognitive imagery styles on the classification performance. To assess any differences between the group of subjects imagining horizontal/vertical directions and the group of subjects imagining diagonal directions we tested mean classification accuracies (across all ROIs) by means of an Mann − Whitney *U* test across the two groups.

### Searchlight analysis

To assess the spatial distribution of brain areas involved in the mental imagery task without restriction to defined ROIs we performed a searchlight analysis (Kriegeskorte et al., 2006). A sphere with a radius of 4 voxels was moved through the cortical ribbon (so that the spheres central voxel always lay within − 1 mm to + 3 mm from the gray/white matter segmentation border) and defined a feature set of 257 voxels (4 voxel radius) that was in turn analyzed with the MVPA pipeline outlined above (without voxel pre-selection and permutation testing). The resulting classification accuracies were tested for significance by means of FDR-corrected Chi-square tests of the confusion matrix and projected onto the inflated surface reconstruction.

Furthermore, we mapped the direction selectivity of these brain areas. A preference map was computed by comparing the four t-value maps of single-direction contrasts and subsequently masked by the significant (p < 0.05) areas in the Chi-square searchlight map.

### Univariate analysis

We performed a general linear model (GLM) analysis for two single subjects that showed very high MVPA decoding accuracies as significant univariate results are expected in such cases. Furthermore, significant univariate results and corresponding maps provide evidence that the MVPA classifier did not pick up on some artifactual or confounding signal rather than brain activity related to the motion imagery task. We used linear predictors for each experimental condition convolved with a standard two-gamma hemodynamic response function. We then computed contrasts for each of the directions of imagined motion against all other directions. We plotted four corresponding t-maps that were thresholded at q < 0.05 (FDR corrected) on the inflated cortical surfaces.

## Results

### Behavioral data

**Fig. 3:**
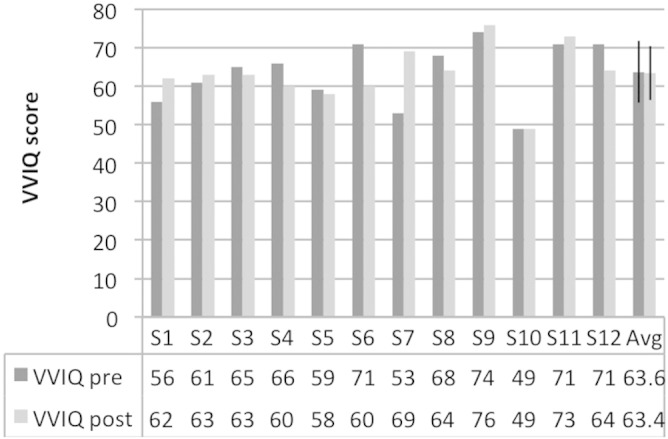
Visual Imagery Questionnaire (VVIQ) scores. VVIQ scores from before the training (pre) and after the scanning session (post) are shown for all subjects (S1 through S12). The last column shows the group averages (Avg); error bars indicate one standard deviation.

**Fig. 4:**
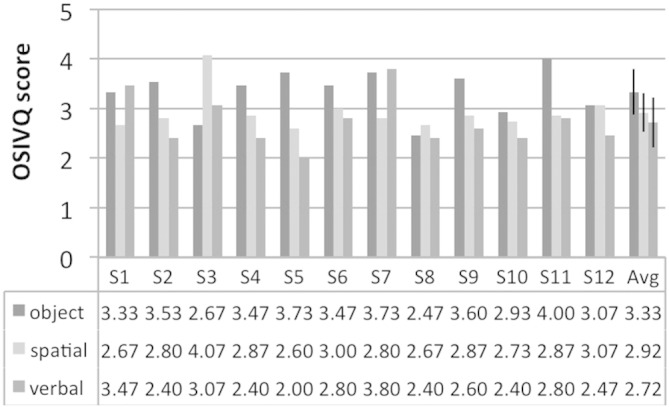
Object-Spatial Imagery and Verbal Questionnaire (OSIVQ) scores. OSIVQ scores for the three different scales “object”, “spatial”, and “verbal” are shown for all subjects (S1 through S12). The last column shows the group averages (Avg); error bars indicate one standard deviation.

VVIQ scores for all subjects are shown in Fig. 3. Scores did not change significantly between before the training and after the scanning session (paired *t*-test; t(11) = 0.12; p = .903). OSIVQ scores on the three scales for all subjects are shown in Fig. 4. The strategies initially reported by subjects are shown in Table 1. In the third training session most subjects reported to not use particular strategies anymore but visualize the reference stimulus directly.

**Fig. 5:**
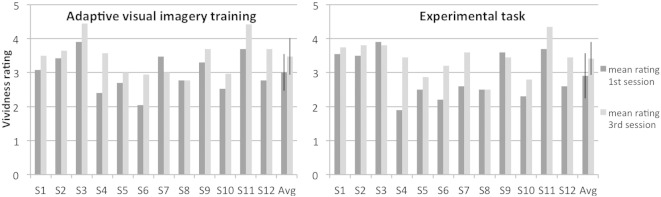
Mean vividness ratings for adaptive visual imagery and experimental task in training sessions. Mean ratings for the vividness of imagery (1: “No image at all, you only ‘know’ that you are thinking of the object” to 5: “Perfectly clear and vivid as normal vision”) are shown for all subjects (S1 through S8) for the first and the last training session in the adaptive visual imagery training (left) and the experimental task (right). The last columns show the group averages (Avg); error bars indicate one standard deviation.

The training sessions improved the subjective ratings for the vividness of the imagery in most subjects (see Fig. 5). On group level, a paired *t*-test between behavioral data from the first and the last training session revealed a significant improvement for the ratings in the adaptive visual imagery training(t(11) = -3.64; p = .004) and the experimental task (t(11) = -3.45; p = .005). Data from the ratings given during the scanning sessions were unfortunately lost due to a software malfunction and, hence, could not be used for data analysis.

### Multi voxel pattern analyses

**Fig. 6:**
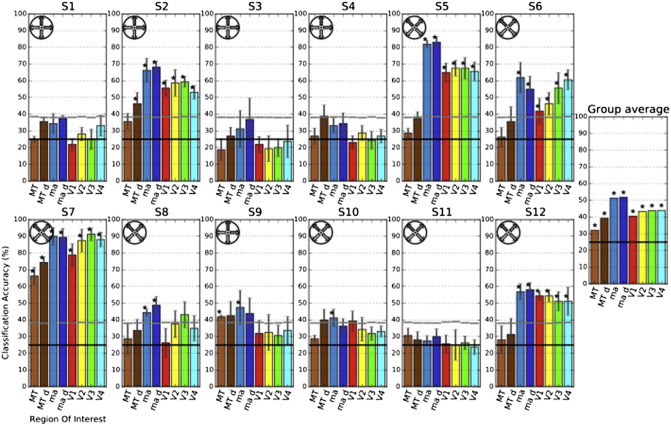
Classification accuracies for imagery of visual motion in four different directions. Average classification accuracies across cross-validations for all subjects (S1–S12) are shown in eight different ROIs. Error bars show one standard deviation of the accuracies' distribution across cross-validations. The black horizontal lines represent the chance level (25%); the gray horizontal lines show the 95th percentile of each 1000-fold permutation test. Asterisks in single subjects indicate significant accuracies (p < 0.05) as assessed by 1000-fold permutation testing. Asterisks in the group average indicate significant accuracies as assessed by Wilcoxon Signed-Rank tests against the average classification accuracy of 1000-fold permutation testing. Orientation wheels at the right show the directions of motion that were imagined by the respective subjects in each row. MT d: 20-fold dilated MT ROI; ma: *visual motion*-*responsive areas* ROI; ma d: 10-fold dilated *visual motion*-*responsive areas* ROI.

Volumes of all defined ROIs are shown in Table 2. Group-level statistics revealed significant classification accuracies for all defined ROIs (MT (W = 9; p = .019), dilated MT (W = 0; p = .002), *visual motion*-*responsive areas* (W = 0; p = .002), dilated *visual motion*-*responsive areas* (W = 0; p = .002), V1 (W = 12; p = .034), V2 (W = 5; p = .008), V3 (W = 6; p = .01), and V4 (W = 3; p = .005)). The single-subject level MVPAs revealed significant classification accuracies for two subjects in the MT ROI, for two subjects in the dilated MT ROI, for five subjects in the V1, V2, V3, and V4 ROIs, for six subjects in the dilated *visual motion*-*responsive areas*, and for eight subjects in the *visual motion*-*responsive areas* ROIs, respectively (see Fig. 6). In subject 7 classification accuracies of up to 91,25% (V3 ROI; chance level at 25%) were observed. Mean classification accuracies (across all ROIs) did not differ significantly between the group of subjects imagining horizontal/vertical motion directions and the group of subjects imagining diagonal motion directions (U = 10; p = .128).

### Correlations with Object-Spatial Imagery and Verbal Questionnaire

**Fig. 7:**
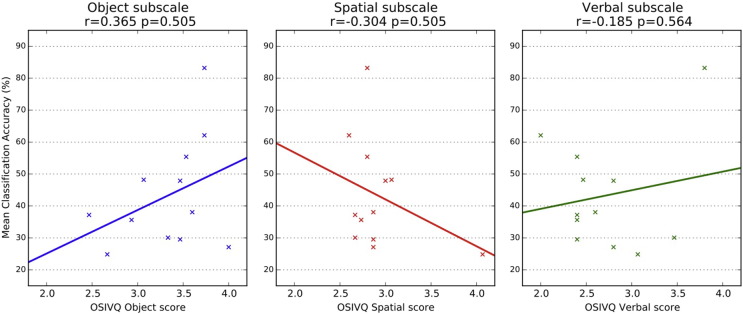
Scatter plots for Object-Spatial Imagery and Verbal Questionnaire (OSIVQ) scores and average classification accuracies (across all ROIs). Scores from the three OSIVQ scales “object” (blue), “spatial” (red), and “verbal” (green) are plotted against the average classification accuracies for each subject. The colored lines show linear regressions for each OSIVQ scale. Correlations and corresponding p-values are shown in titles of each subplot.

No correlation between OSIVQ scores and classification accuracies was significant (r_Object_ = .365 (p = .243); r_Spatial_ = −.304 (p = .337); r_Verbal_ = -.185 (p = .564); see Fig. 7).

### Searchlight analysis

**Fig. 8:**
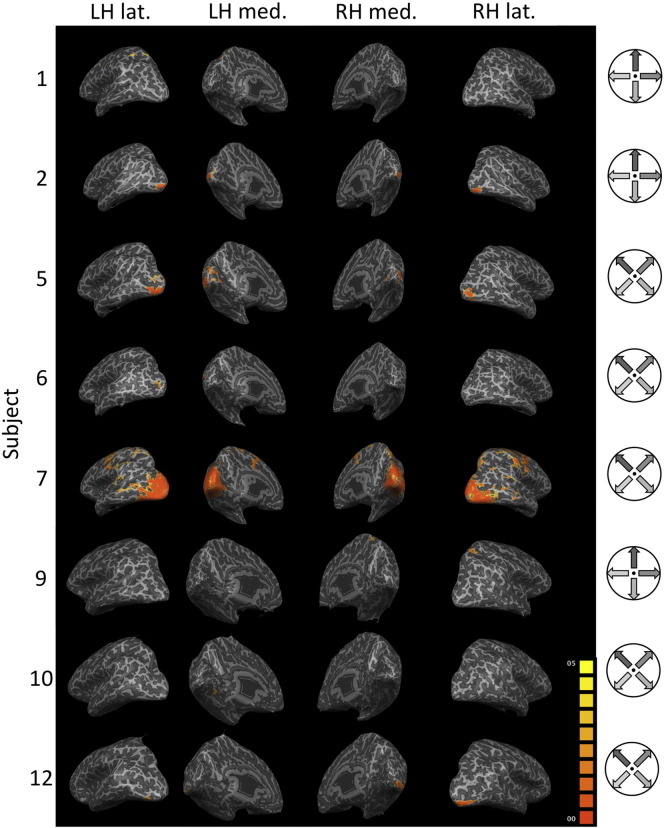
Searchlight maps for eight subjects. Significant areas in the searchlight map (p < 0.05 FDR corrected; cluster-thresholded at 50 mm^2^) in the two inflated hemispheres of subjects 1, 2, 5, 6, 7, 9, 10, and 12 (there were no significant patches on the remaining subjects). Warmer colors (orange > yellow) indicate lower p-value. On the right the motion directions that had to be imagined are shown for each subject. LH: left hemisphere; RH: right hemisphere; lat.: lateral view; med.: medial view.

**Fig. 9:**
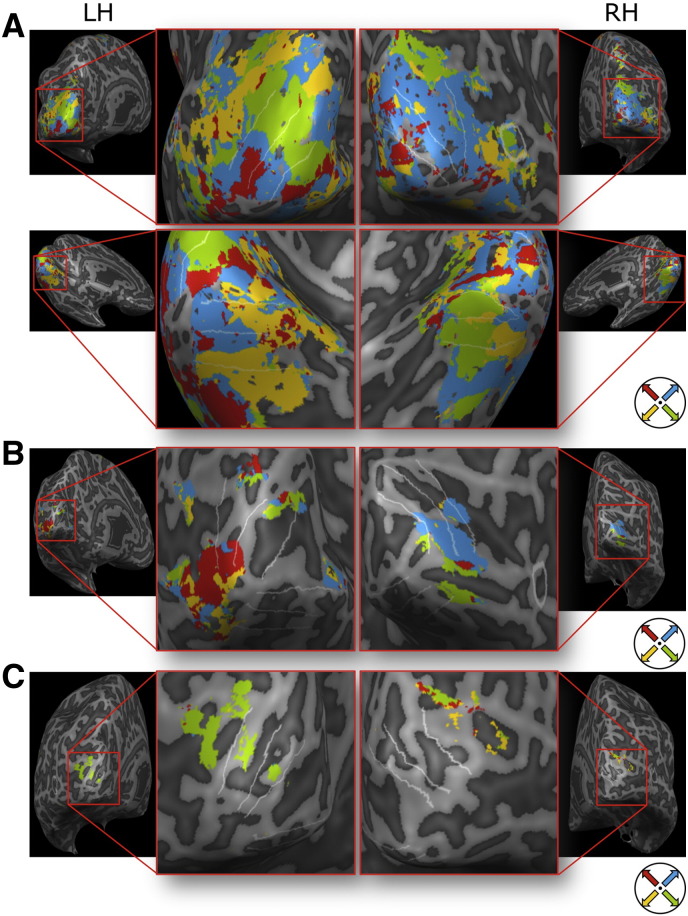
Searchlight-thresholded univariate preference maps for three subjects. Preference maps for (A) subject 7, (B) subject 5, and (C) subject 6 calculated from univariate contrasts for each imagined motion direction are shown for those areas exhibiting significant effects in the searchlight map (p < 0.05 FDR corrected). Colors indicate preferred imagined motion directions and correspond to the direction wheel at the bottom right. Areas V1 through V4 are delineated with white lines. The MT ROI is delineated in white (white circular delineation only visible on the magnified right hemispheres of subjects 7 and 5).

Maps showing cortical areas with significant searchlight accuracies showed inter-individual differences (see Fig. 8). In subject 7 large parts of the early visual system showed significant decoding accuracies (see Fig. 9A). In subject 5 mainly V2, V3, and V4 areas going into the foveal confluence showed significant decoding accuracies (see Fig. 9B). In subject 6 areas V2L, V3L, and V4L (and beyond) showed significant decoding accuracies in the left hemisphere. In the right hemisphere area V4L showed significant decoding accuracies (see Fig. 9C). In subject 8 a superior parietal region showed significant decoding accuracies.

Preference maps revealed individually distinct patterns. In subject 5 and 7 a difference in preference from central to peripheral parts of the visual field (following the eccentricity tuning) was observed.

### Univariate analysis

**Fig. 10:**
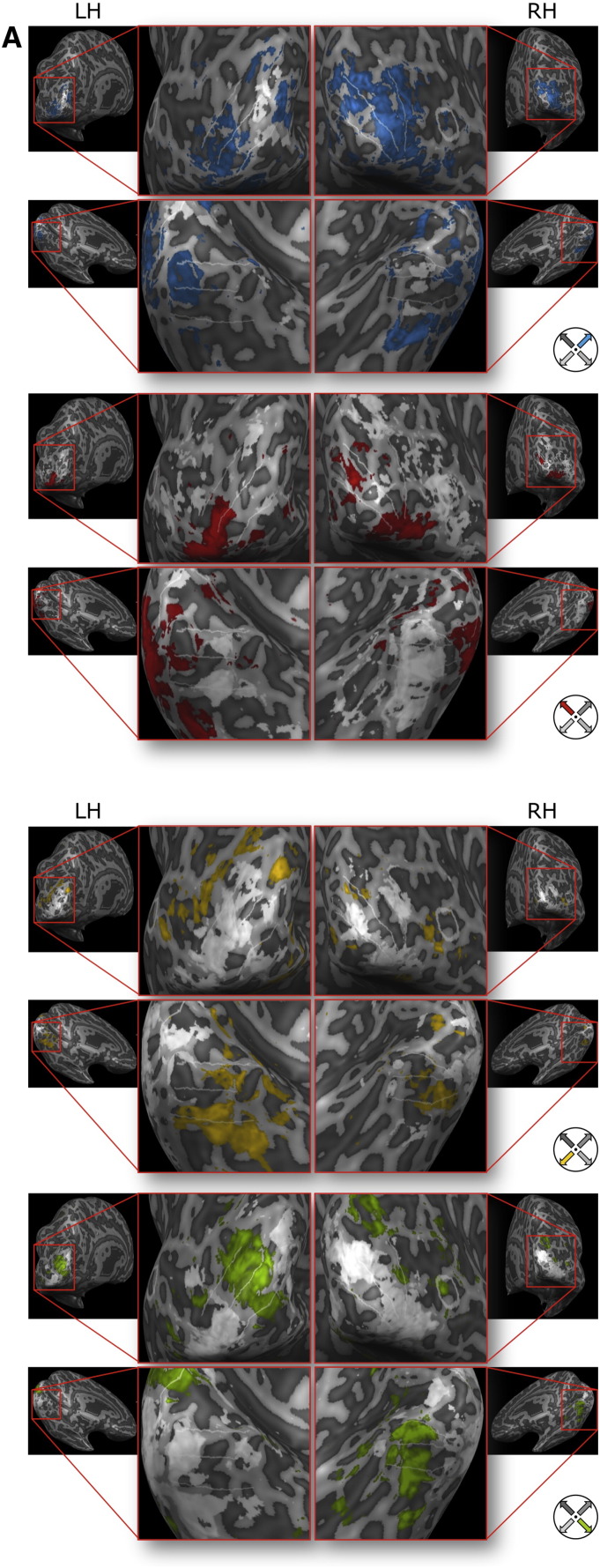

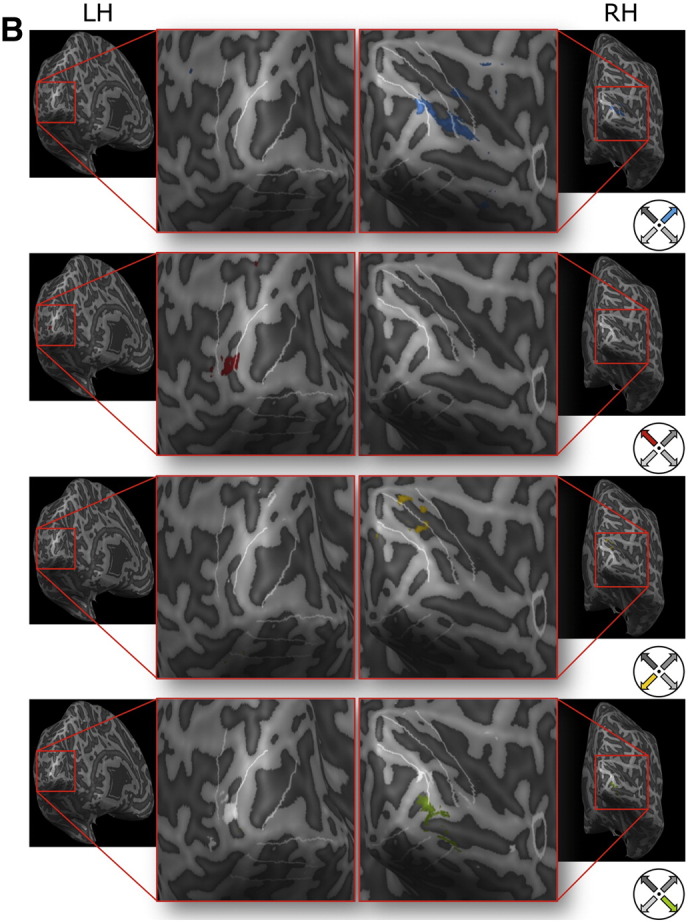
Univariate single-direction contrast maps for two subjects. T-maps (one direction vs all other directions) for (A) subject 7 and (B) subject 5 for each imagined motion direction are shown (q < 0.05; FDR corrected). Colors correspond to the direction wheel at the bottom right. Gray/white map colors correspond to negative t-values. Areas V1 through V4 are delineated with white lines. The MT ROI is delineated in white (white circular delineation only visible on the magnified right hemispheres of subject 7 and 5).

For subject 5 and 7 single-subject GLM analyses showed significant activations for the different directions of imagined motion. Fig. 10 shows corresponding t-maps thresholded at q < 0.05 (FDR corrected) for the same areas on the inflated cortex as in Fig. 9A and B.

### Eye-tracking data

**Fig. 11:**
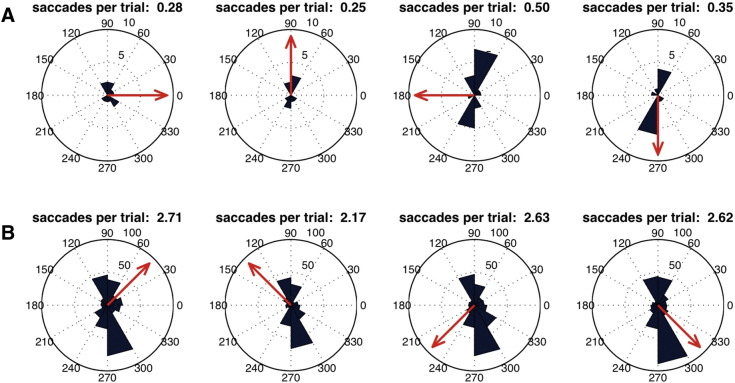
Eye-tracking data from (A) one subject imagining horizontal/vertical directions (subject 9) and (B) three subjects imagining diagonal direction (subjects 10, 11, and 12). For (A) and (B) histograms of saccade directions during trials are plotted for all four different directions of imagined motion (indicated by red arrows). The number of saccades per trial is shown above each polar plot.

No systematic eye movements associated to the different motion imagery directions were observed in subject 9 through 12 (see Fig. 11). The test for equal median directions was not significant for any subject (S9: P(3) = 1.159 p = .763; S10: P(3) = 2.018 p = .569; S11: P(3) = 0.471 p = .925; S12: P(3) = 0.352 p = .95), that is, the saccade directions did not differ significantly between different imagined motion directions. The correlation between the direction of imagined motion and the saccade direction was also not significant for any subject (S9: r = − .124 p = .37; S10: r = .008 p = .878; S11: r = .049 p = .32; S12: r = − .02 p = .666). Furthermore, the analyses of the raw eye gaze position did neither return any significant result in the test for equal median directions (S9: P(3) = 2.514 p = .473; S10: P(3) = 7.118 p = .068; S11: P(3) = 4.48 p = .214; S12: P(3) = 4.571 p = .206) nor in the test for circular–circular correlations (S9: r = .018 p = .83; S10: r = .047 p = .56; S11: r = .04 p = .626; S12: r = .046 p = .543).

## Discussion

Using high-resolution imaging at 7 T, we were able to successfully decode directions of imagined visual motion in fMRI data recorded from the visual system. Without any visual stimulation, subjects were able to activate their visual system so specifically that neuroimaging data recorded from areas normally activated during visual perception could predict the imagined motion direction.

### High sub-categorical decoding accuracies

The decoding accuracies ranged from non-significant results in four subjects to accuracies of up to 91,25% (4-class classification) in a single subject — a level rarely reached even in studies decoding perceived motion.

### Localization of decodable information

Apart from the more global *visual motion*-*responsive areas* and dilated *visual motion*-*responsive areas* ROIs, the best classification accuracies were achieved in the V3 and V4 ROIs. These seem to be predominant areas to decode direction of motion during imagery. The results from the searchlight analysis complement these findings. Decoding with significant accuracies was mainly possible in the areas targeted by the ROI analyses and more pronounced in areas V3 and V4. More than that, the results from the searchlight analysis supported the local and potentially fine-grained origin of information in our study. Relatively local brain activation patterns — the searchlight sphere had a radius of 4,4 mm (4 voxels) — predicted the imagined motion direction with substantial accuracy (up to 83,75% in subject 7). These high decoding accuracies in single spheres might hint to a fine-grained columnar organization of motion-selective cortical areas. Though there is an ongoing debate on whether MVPA is able to pick up patterns of brain activation at a spatial scale beyond the recorded spatial resolution (Carlson, 2014, Op de Beeck, 2010a, Op de Beeck, 2010b) and direction preferences in early visual areas seemed to have a rather coarse pattern, the classifiers might have picked up on a more fine-grained organization of direction-selective columns because of a biased sampling (Haynes and Rees, 2006).

### Decoding in hMT +

Though the information encoded in localized MT voxels gave good classification accuracies in some subjects, optimal results were only achieved when voxels from earlier visual areas were included. This is in line with previous results revealing that the decoding of the direction of perceived visual motion achieves higher accuracies when analyzing data from V1 through V4 than from hMT + (Kamitani and Tong, 2005). Moreover, recent work by Wang et al. (2014) showed that the decoding of motion direction during perception is mainly driven by an “aperture-inward” response bias producing good classification accuracies in V1, V2, and V3, but not hMT +. Although we recorded at a higher spatial resolution and decoded imagined visual motion this bias might account for the high classification accuracies in early visual areas and low classification accuracies in hMT +.

With a functional spatial resolution of 1.1 mm (iso-voxels) we are close to the resolution needed to image columnar structures. In hMT + for instance, axis-of-motion columns are estimated to have a width of 2–2.8 mm (Zimmermann et al., 2011). A columnar organization of hMT +, where neighboring columns prefer motion directions that are opposite (*i*.*e*. 180° difference) to form axis-of-motion columns, was proposed before (*e*.*g*., Born and Bradley, 2005). Such an organization, however, might have actually impaired decoding in hMT + in our study as we recorded with a spatial resolution that might just fall short of capturing single direction-of-motion columns. In order to reveal the precise organization of direction-selective regions in early visual areas and hMT +, an even higher spatial resolution has to be reached by employing, for example, spin echo sequences that seem to be even more sensitive to local activations than gradient echo sequences (De Martino et al., 2013, Uğurbil et al., 2003, Uludağ et al., 2009, Zimmermann et al., 2011).

### Univariate results

When looking at the univarite statistical maps for subject 7 and 5 we observed topographic effects in mainly early and mid-level visual areas. The t-maps for each direction of imagined motion (one-vs-all) show an alignment to borders between early visual areas and to different eccentricities (foveal *vs*. peripheral). While there is no clear organization of this topography the significant patches overlap with the individual thresholded searchlight accuracy maps. Individual topograhpies might be influenced by the individual imagery style and strategy.

### Inter-individual differences

We found no significant correlations between decoding accuracies and any questionnaire scores. Though the subscales of the OSIVQ showed differential correlations with decoding accuracies none of these correlations became significant. It would be important to explore such correlations in bigger sample sizes to get further insight into the possible reasons for inter-individual differences during imagery decoding. This could potentially explain the mixed results in other imagery decoding studies (Kaas et al., 2010, Klein et al., 2000, Knauff et al., 2000). It has to be emphasized that the sample on which our results are based is of limited size so that we cannot rule out small or medium effects that would only be observable in bigger sample sizes.

### Limitations and outlook

Due to the inherently private nature of imagery, the internal “stimulation” in this experiment was hard to control, potentially inducing additional variability. However, we argue that our findings are based on visual mental imagery.

Most importantly, we decoded the direction of motion in visual areas of the cortex, thereby ruling out many confounding sources of decodable information. Furthermore, simple physiological changes like motion artifacts or changes in breathing are too unspecific to produce the effects of the observed magnitude within the complex 4-class-classification design. Cross-modal influences on the visual system could, potentially, lead to decodable information in early visual areas. Vetter et al. (2014) give an example for cross-model influences; they showed that the content of auditory stimulation can be decoded in early visual areas. In line with this result, one could argue, that the classification results in our study are driven solely by the different auditory cues preceding the imagery trials. However, the motion imagery in our study presumably was a visuospatial imagery task with a high cognitive load (subjects reported that it would need a high cognitive engagement to maintain the continuous mental image of moving dots). In the study by Vetter and colleagues, the addition of such a visuospatial imagery task to their original auditory stimulation eliminated most of the cross-modal influences. Therefore, we do not expect our results to be merely based on the auditory cue. Moreover, the achieved classification accuracies in our study are much higher than the classification accuracies reported in Vetter et al. (2014) and, thus, likely not only an effect of the auditory cue.

It could also be argued that the decoding of motion imagery might merely be an artifact of eye movements (see also Laeng and Teodorescu, 2002). When we analyzed eye-tracking data recorded during the experiment in the fMRI scanner, there were no significant associations between the imagined motion direction and the direction of saccades. Furthermore, previous work show behavioral effects of motion imagery that cannot be explained by eye movements; Winawer et al. (2010) had subjects imagine inward and simultaneously outward moving gratings inducing a motion aftereffect. In this experiment corresponding eye movement accounting for the observed effects, would have been physiologically impossible. In a future study a comparison with a closed-eyes condition would be interesting as other cognititve processes might be involved when bottom–up visual input is missing entirely.

Our results could be explained by a purely attention-based modulation of visual areas. Winawer et al. (2010) argue that top-down attention modulation would only operate on feed-forward inputs to the visual cortex. However, in their study they find equal behavioral effects of mental imagery in closed- and open-eyes conditions, where in the closed-eyes condition feed-forward input is clearly missing. Even top-down modulations would still be in line with the hypothesis that imagery tasks use neural circuitry also involved in perception tasks. Our results could be caused by an internally produced stimulus that activates early visual cortex, and an attentional modulation based on the direction of imagined motion.

Finally, neural patterns underlying mental imagery have also been shown in frontal brain regions (*e*.*g*., Ganis et al., 2004). Tailoring the fMRI sequence to optimally record from visual areas with a high spatial resolution, however, we were not able to record data from the frontal cortex.

### Conclusions and implications

Our results are of remarkable significance in single subjects and show that it is possible to reliably decode the content of complex visual imagery from neuroimaging data in single trials. Sub-categorical decoding of visual mental imagery (not ‘if’ but ‘what’) is not only relevant to advance neuroscientific knowledge, but would also enable advanced brain-computer interfaces (BCI; for reviews see Kübler and Neumann, 2005, Birbaumer and Cohen, 2007, Goebel et al., 2010, Nicolas-Alonso and Gomez-Gil, 2012). In a typical BCI setup (*e*.*g*., Sorger et al., 2012), the user is asked to perform a mental imagery tasks (*e*.*g*., imagine to move the hand *vs*. imagine to sing a song) that corresponds to different information entities that the user wants to encode (*e*.*g*., to steer the cursor on the screen to the left *vs*. to the right). The BCI then classifies these different categories of mental imagery tasks based on the acquired brain activation data and executes the associated intended actions (*e*.*g*., steering the cursor to the right). However, the possibility of sub-categorical decoding would allow expressing intentions in a much more natural way; the imagery of rightward visual motion (instead of imagining singing a song) could make a cursor on the screen go to the right. The recent trend towards explicit models of representation (Naselaris and Kay, 2015) would add to a closer connection between imagery content and intention. Though the use of BCIs can be trained (Wolpaw et al., 2002), especially novice users rely on a close connection between imagery and translated action and would most probably benefit from such an advanced setup. In order to achieve high and stable single-trial classification accuracies that are necessary for BCIs, it will be interesting to employ spin echo sequences with an even higher spatial resolution in the future. On a sub-millimeter functional resolution columnar-level spatial separation of direction-of-motion features might become more explicitly exploitable.

## Conflict of interest

The authors declare no competing financial interests.

## Figures and Tables

**Table 1:** Strategies used by subjects as reported during the first training session.

Subject	Strategy
1	Upwards: bubbles rising in a glass; downwards: snow falling (blizzard); other two directions: city lights viewed from an airplane
2	Downwards: rain; no other specific strategy
3	Up/down: tetris game; left/right: tennis match
4	Rain/bubbles while visualizing the aperture to have a guidance for the imagery
5	Swimming tadpoles
6	No particular strategy
7	No particular strategy
8	Focus on the dot pattern; no other particular strategy
9	Upwards: bubbles rising in a glass; downwards: snow falling; left/right: buffalo herd running
10	Try to keep the almost faded out dots in mind
11	Try to keep the almost faded out dots in mind
12	Upwards: water hose; downward: rain

**Table 2:** Volumes of defined Regions of interest (ROIs).

Subject	MT	MT d	ma	ma d	V1	V2	V3	V4
1	1026 (1.366)	8496 (11.308)	20958 (27.895)	40373 (53.736)	3343 (4.45)	4087 (5.44)	3173 (4.223)	2900 (3.86)
2	914 (1.217)	7497 (9.979)	32198 (42.856)	53566 (71.296)	4489 (5.975)	4541 (6.044)	4216 (5.611)	3621 (4.82)
3	1161 (1.545)	8404 (11.186)	28763 (38.284)	58326 (77.632)	5014 (6.674)	4315 (5.743)	4388 (5.84)	3334 (4.438)
4	1290 (1.717)	9344 (12.437)	27410 (36.483)	53552 (71.278)	2807 (3.736)	3708 (4.935)	3370 (4.485)	3076 (4.094)
5	1023 (1.362)	7584 (10.094)	41955 (55.842)	71583 (95.277)	4547 (6.052)	5049 (6.72)	4278 (5.694)	3678 (4.895)
6	986 (1.312)	8105 (10.788)	35565 (47.337)	63736 (84.833)	3808 (5.068)	3746 (4.986)	4088 (5.441)	3713 (4.942)
7	998 (1.328)	8145 (10.841)	38272 (50.94)	72269 (96.19)	5210 (6.935)	6244 (8.311)	6497 (8.648)	4535 (6.036)
8	1160 (1.544)	8486 (11.295)	23668 (31.502)	53451 (71.143)	3936 (5.239)	5032 (6.698)	4220 (5.617)	2912 (3.876)
9	990 (1.318)	8473 (11.278)	43215 (57.519)	75581 (100.598)	3596 (4.786)	3135 (4.173)	3034 (4.038)	3478 (4.629)
10	1013 (1.348)	8458 (11.258)	20816 (27.706)	42934 (57.145)	3820 (5.084)	3492 (4.648)	3016 (4.014)	2567 (3.417)
11	1003 (1.335)	7670 (10.209)	22128 (29.452)	44265 (58.917)	4226 (5.625)	3910 (5.204)	3431 (4.567)	3417 (4.548)
12	1012 (1.347)	8367 (11.136)	21256 (28.292)	52395 (69.738)	4500 (5.99)	3364 (4.477)	3163 (4.21)	3347 (4.455)
Avg	1048.0 (1.395)	8252.417 (10.984)	29683.67 (39.509)	56835.92 (75.649)	4108.0 (5.468)	4218.583 (5.615)	3906.167 (5.199)	3381.5 (4.501)
Std	98.825 (0.132)	484.973 (0.645)	8028.314 (10.686)	11298.38 (15.038)	663.526 (0.883)	846.353 (1.126)	933.923 (1.243)	483.801 (0.644)

Numbers of voxels (voxel size = 1.1 × 1.1 × 1.1 mm^3^) for all defined ROIs are shown for all subjects. In parentheses volumes are shown converted to (rounded) cm^3^. MT d: 20-fold dilated MT ROI; ma: *visual motion*-*responsive areas* ROI; ma d: 10-fold dilated *visual motion*-*responsive areas* ROI. Avg: Average (across subjects); Std: Standard deviation (across subjects).
